# A 25 years experience of group-housed sows–reproduction in animal welfare-friendly systems

**DOI:** 10.1186/1751-0147-56-37

**Published:** 2014-06-09

**Authors:** Stig Einarsson, Ylva Sjunnesson, Fredrik Hultén, Lena Eliasson-Selling, Anne-Marie Dalin, Nils Lundeheim, Ulf Magnusson

**Affiliations:** 1 Division of Reproduction, Department of Clinical Sciences, Swedish University of Agricultural Sciences, SE-750 07 Uppsala, Sweden; 2 Medical Product Agency, PO Box 26, SE-751 03 Uppsala, Sweden; 3 Swedish Animal Health Service, Kungsängens Gård, SE-753 23 Uppsala, Sweden; 4 Department of Animal Breeding and Genetics, Swedish University of Agricultural Sciences, SE-750 07 Uppsala, Sweden

**Keywords:** Sow reproduction, Housing, Animal welfare, EU regulations

## Abstract

Since January 1 2013, group housing of sows has been compulsory within the European Union (EU) in all pig holdings with more than ten sows. Sows and gilts need to be kept in groups from 4 weeks after service to 1 week before the expected time of farrowing (Article 3(4) of Directive 2008/120/EC on the protection of pigs). The legislation regarding group housing was adopted already in 2001 and a long transitional period was allowed to give member states and producers enough time for adaptation. Even so, group housing of sows still seems to be uncommon in the EU, and is also uncommon in commercial pig farming systems in the rest of the world. In this review we share our experience of the Swedish 25 years of animal welfare legislation stipulating that sows must be loose-housed which *de facto* means group housed. The two most important concerns related to reproductive function among group-housed sows are the occurrence of lactational oestrus when sows are group-housed during lactation, and the stress that is associated with group housing during mating and gestation. Field and clinical observations in non-lactating, group-housed sows in Sweden suggest that by making basic facts known about the pig reproductive physiology related to mating, we might achieve application of efficient batch-wise breeding without pharmacological interventions. Group housing of lactating sows has some production disadvantages and somewhat lower productivity would likely have to be expected. Recordings of behavioural indicators in different housing systems suggest a lower welfare level in stalled animals compared with group-housed ones. However, there are no consistent effects on the reproductive performance associated with different housing systems. Experimental studies suggest that the most sensitive period, regarding disturbance of reproductive functions by external stressors, is the time around oestrus. We conclude that by keeping sows according to the pig welfare-friendly Directive 2008/120/EC, it is possible to combine group-housing of sows with good reproductive performance and productivity. However, substantially increased research and development is needed to optimize these systems.

## Introduction

On January 1 2013, group housing of sows became compulsory in the European Union (EU) for animal welfare reasons. The Directive making group housing of sows compulsory within the EU in all pig holdings with more than ten sows stipulates that sows and gilts are to be kept in groups from 4 weeks after service to 1 week before the expected time of farrowing (Article 3(4) of Directive 2008/120/EC on the protection of pigs [1]). The legislation regarding group housing was adopted already in 2001 and a long transitional period was allowed to give EU member states and producers enough time for adaptation. Still, estimation based on data from the member states sent to the EU Commission in March 2012 indicates that considerable efforts are needed to reach compliance to the Directive for some of the member states that are major pig producers within the EU [2]. This is not only an animal welfare issue, but it will also create a distortion of the competition among producers since the transition to group housing is associated with costs, and the group-housing per se may imply lowered productivity.

A recent inventory of the legislation for housing of pigs in the EU showed considerable differences between countries [3]. Only a few countries like Sweden, UK and the Netherlands had legislation demanding loose housing of sows. Worldwide, pig-housing systems are diverse. In the US where there is a strong intensification related to industrialized production of livestock, more than 80% of sows are estimated to be kept individually confined throughout pregnancy [4]. In the emerging economies in Asia showing the world’s most rapid increase in pig production, the majority of pigs are still kept in small-scale production systems sometimes including free-ranging systems [5]. However, in China, the world’s largest producer of pork, where currently only 10% of the pork originates from large-scale commercial farms, there has been a rapid expansion of holdings where sows are kept confined in the same way as in most European countries and the US [6,7]. The same pattern of expansion of the pig sector can be seen in South East Asian countries [8].

In Sweden, new animal welfare legislation was introduced in 1988 [9] prohibiting fixation of sows. Sows have to be kept loose during lactation, after weaning and during pregnancy, i.e., during the entire reproductive cycle. Fixation for a short period of time is allowed in cases where this is considered necessary to ensure piglet welfare, or in case of illness. In this review we share the Swedish experience of 25 years of this animal welfare legislation that stipulates that sows must be loose-housed; which have had as a consequence that Swedish sows to a large extent has been group-housed ever since. Here we discuss housing solutions that are based on the sow’s physiology and natural behaviour as well as on remaining challenges regarding production results and reproductive performance.

## Review

### The natural behaviour of pigs

It is evident that the behaviour pattern of the ‘modern sow’ to large extent still resembles that of the wild boar [10-12]. Typically the wild boar forms small groups of females, consisting of between two and five adult individuals with offspring. By contrast, the males keep isolated or form bachelor groups, which associate with the female group at times when sows are sexually receptive [13-16]. Like the wild boar, a domesticated, free-ranging sow allowed to express her natural behaviour isolates from the group for some weeks around farrowing and subsequently returns to the female group [11,12]. Under these conditions, weaning is a gradual process in which the contact between the sow and her offspring continuously decreases. This eventually releases the neuroendocrine block to reproductive functions and the sow returns to cyclic activity [17-19]. This contrasts to the situation for individually housed sows where suckling intensity is maintained and the lactation period limited and therefore ovulation very rarely occurs during lactation [20,21].

### Concerns related to reproduction

Housing conditions that aim to allow the sow to express her natural behaviour could counteract the efforts to maintain adequate management routines due to the fact that the reproductive functions are affected as outlined above. For instance oestrus during lactation in group-housed sows during lactation leads to increased variation in the weaning-to-mating interval, which in turn makes batch-wise management practice problematic [22,23]. Notably, it is well established that batch-wise management of sows, i.e., placing all sows in the farrowing unit at the same time, and taking all sows out of the farrowing unit at weaning, followed by cleaning and disinfection, has a major biosecurity advantage [24].

Increasing group size, as compared with keeping the size of naturally formed groups, which is necessary from a management perspective, may also cause problems. As previously mentioned, the female pigs form family units of one or several sows and their offspring [15], and the individuals in the family unit avoid contact with other, unfamiliar sows [25]. In commercial group-housing systems, however, mixing of unfamiliar sows is difficult to avoid. When lactating sows are housed individually, grouping of unfamiliar sows usually takes place at least once after weaning. This enforces social re-orientation where dominance hierarchy needs to be established, which inevitably causes aggressive behaviour. This affects not only the animals’ welfare but may also affect their reproductive performance. Consequently, there are reports indicating that impaired reproduction in group-housed sows is a growing problem in many herds [26].

The two most important concerns related to reproductive function among group-housed sows are: the occurrence of lactational oestrus when sows are group-housed during lactation; and the stress that is associated with group housing during mating and gestation.

### Swedish pig housing systems

#### ***Group housing of lactating sows***

Various types of group housing for lactating sows have been developed and scientifically evaluated during the last 50 years [27] and it has become evident that onset of oestrus is highly variable in group-housing conditions. Experimental and observational studies have shown that apart from a decrease in suckling intensity in these systems [28], several other factors such as boar contact and feeding intensity are correlated to return to oestrus during lactation [29,30]. The idea of stimulating the sows to return to oestrus and be mated during lactation, as a means of improving production output, was, however, soon abandoned as the disadvantages connected to this became evident. To allow mating throughout the lactation period is difficult to combine with the effective control of transmission of infections in the batch-wise production concept [31,32].

However, the group-housing system for lactating sows has survived in Sweden, mainly in commercial farms certified as organic. Both outdoor and indoor systems exist and in both, the sows are often kept isolated at farrowing and mixed with other sows a few weeks later [27,33]. On these farms, ovulation, detected through repeated monitoring of progesterone metabolite excretion in faeces [34], occurs to varying extent before weaning. It has been noted that older, multiparous sows (five parities or more) are more prone to return to cyclic activity [22,23], likely due to a more rapid weaning process among them [28].

Piglet growth appears not to be affected by group housing as compared with conventional single-housing systems, while a higher pre-weaning mortality rate has been noted among group-housed multiparous sows, which is likely related to an accelerated weaning process among older sows, as previously mentioned [35]. The latter is supported by findings in a recent field study, where there was a tendency to a lower number of weaned pigs per litter in farms where sows were kept in groups compared with farms where the sows where kept individually [36].

#### ***Group housing of non-lactating sows***

Different systems for group housing during the mating and gestation period have been developed since Sweden adopted the animal welfare legislation prohibiting fixation of sows. A common practice for the period after weaning until insemination and/or until pregnancy testing is to keep the sows grouped on deep litter straw bedding, allowing for individual feeding in lockable feeding stalls. After this period, from mating until farrowing, the sows are often kept in larger groups until farrowing when they are moved to the farrowing areas.

During the 1990s, parallel to the development of the new management systems for loose-housed sows, the all-in-all-out systems for fatteners became more common in pig production in Sweden (see Table 1). This made it necessary to breed and subsequently wean a large number of sows within a short time.

**Table 1:** Changes in pig production systems in Sweden during the last 30 years (Data provided by the Swedish Animal Health Service)

**Change**	**Consequence**
Gradually altered herd structure and sow number	As in many other countries in Europe, the herd structure in pig production in Sweden has changed drastically over the last 30 years. In 1980, there were 19 000 holdings with a mean of 13 sows per herd and 240 000 sows in total; in 2012, the corresponding figures were 750 holdings, 165 sows per herd and 125 000 sows in total.
Ban on antimicrobial growth promotors, 1986	Following the Swedish ban on antimicrobial growth promoters in 1986 there was a transition in pig production, from a continuous production concept into batch-wise (all-in-all-out) concepts to maintain good animal health. Based on the all-in-all-out concept, large groups of sows are weaned and bred at the same time, which in turn has increased the use of artificial insemination (AI) to about 95% of all services in 2013
Ban on dry sows in stalls, 1988	Following the ban on dry sows in stalls legislation in 1988, the sow pool concept, i.e. breeding herds with satellite nursing herds, was introduced to reduce the need for rebuilding housing facilities. In 2013, there were 19 sow pool units in Sweden, with approximately 20% of all Swedish sows.

Artificial insemination is the predominant practice for breeding sows in Sweden (approximately 95% of the sows are subjected to AI, Wallgren M, personal communication). Reproductive hormones for induction of oestrus is not used in Sweden, as it has been found unnecessary in the management system used [37]. In these systems careful attention is paid to the gilts’ and sows’ oestrous behaviour and the scientific knowledge regarding the factors influencing this behaviour is used [38-40]. These factors include the interaction between the sows within the group, as well as the interaction between the teaser boar and the sows. They also include some positive stress, which stimulates induction of oestrus, as shown in gilts [41] (see also below).

In the vast majority of the housing systems used in Sweden today, the weaned sows are moved to a special breeding area. During the first 1–2 days they express dominance and subordinate behaviour to establish a dominance hierarchy within the group [42]. It is important that there should be enough space during this stressful period to avoid prolonged fighting, which could cause depression of oestrous symptoms [43]. However, a certain degree of stress may be positive [41], as it will contribute to inducing oestrus as long as the sows are able to cope with it. Therefore, provided sufficient space is allowed, the mixing of the sows will most often have a positive influence on the return to cyclic activity. The weaning-to-oestrus interval is approximately 4–5 days in this system, depending on the parity and condition of the sow [44]. Anoestrus is a rare event among group-housed sows [45], but it may be difficult to detect oestrus due to the hierarchy among the individuals in the group [43]. Boar contact is the strongest stimulus to induce the standing reflex [46]. Therefore, the sow should be able to hear and smell the boar from the time of weaning. However, nose-to-nose contact with the boar should be postponed to the moment of oestrous detection (control of standing reflex) and insemination. This is accomplished in a specially designed area close to the boar pen and ideally with a small number, 2–3, of sow at one time [37].

#### ***Production results in Swedish systems***

**Figure 1:**
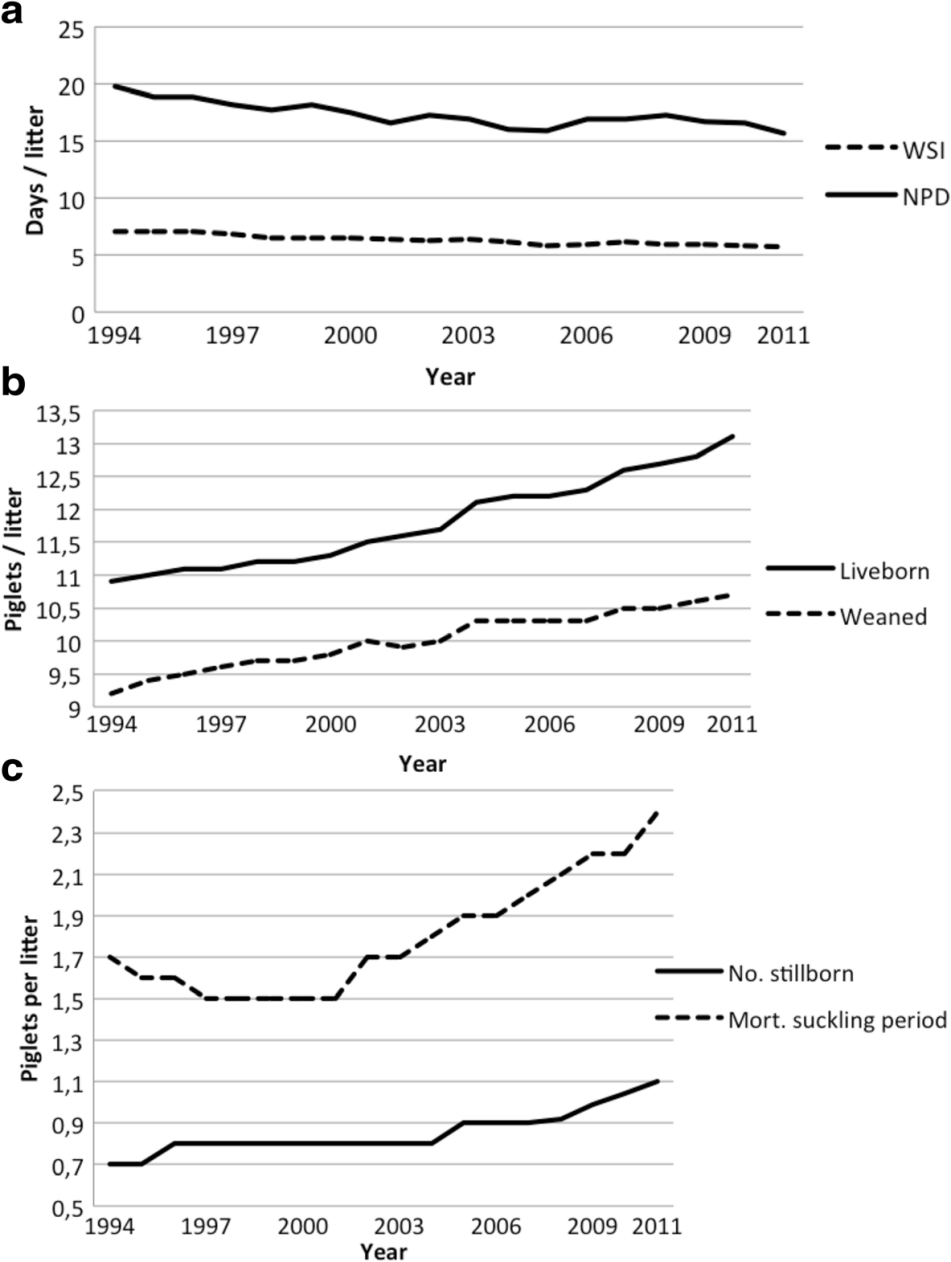
**Phenotypic trends in Swedish piglet production, based on data from the herd monitoring program PigWin (Svenska Pig, 2013 [**[47]**]). a)** Weaning to service interval (WSI) and number of non productive days (NPD). **b)** Litter size, number of liveborn piglets (Liveborn) and number of weaned piglets (Weaned) per litter. **c)** Piglet mortality, number of stillborn piglets (No. stillborn) and number of piglets died during suckling period (Mort. suckling period) per litter.

No studies on productivity at herd level have been performed in Sweden before and after, or during, the successive changes to the housing systems. Consequently, information is lacking regarding which effect these changes have potentially caused. However, an increasing proportion of the piglet-producing herds in Sweden are using a herd-monitoring software, PigWin [47]. On the basis of information from a selected number of herds, recorded using this software the weaning-to-service interval (WSI) and non-productive days (NPDs) per litter have decreased, NPDs faster than WSI (Figure 1a). Since WSI is included in NPDs, this indicates that the number of “waste days”, including empty sows and sows that are re-mated, is diminishing. The litter size has increased substantially during the last 15 years, but that the gap between the number of liveborn piglets and the number of weaned piglets has increased over time (Figure 1b). This means that that there has been a slight increase in piglet mortality during the suckling period together with the increased stillbirth rate (Figure 1c). Similar national data from countries with confined sows is difficult to get hold on, but the rates in presented here are not exceptional in any direction compared to data in scientific reports from farms in such countries.

### Studies on stress and reproduction in group-housed sows

Several management procedures in modern pig production can act as stressors on the animals. Though they are believed to be more animal-friendly, loose-housed systems also may include stressors. For instance, as previously mentioned, the number of sows in group-housing systems is in most cases much higher than in groups formed in the wild. Also, it is difficult to avoid regrouping of the sows. Establishing a new social hierarchy in a limited space involves aggressive behaviour among the animals. An animal that cannot cope with this procedure may have reduced wellbeing with impaired reproductive performance. For instance, high-ranking sows in oestrus mount low-ranking sows, which may be stressful for the latter category of sows [43]. These issues warrant research on the relations between housing, stress and reproductive performance in sows.

#### ***Comparisons between different housing systems***

A large descriptive study of about 1300 Finnish sow units reports that re-breeding after an irregular oestrus-to-oestrus interval occurred more often in group-housed sows compared with sows kept in individual stalls, particularly during late summer and autumn [48]. Furthermore, other studies report that group-housing conditions resulted in fewer piglets per litter compared with individual housing [49,50]. Also, group housing may result in impairment of heat detection and response to boar stimulation, especially among low-ranking, first-parity sows [51]. In a comprehensive study by Karlén et al. [52] on welfare including reproduction in gestating sows in conventional stalls and in large groups on deep litter, sows on deep litter had a higher return to oestrus rate after mating. Altogether, the reproductive parameters recorded show that sows in stalls weaned more piglets per mated sow than in large groups. The results suggest that sows in large groups on deep litter face greater welfare challenges in the early stages of gestation, all possibly a consequence of aggression. By contrast, sows in stalls face greater welfare challenges later in gestation based on a higher incidence of foot and leg problems. In addition, the evidence of stereotypical behaviour may indicate disadvantages for sows kept in stalls for the whole gestation. On the other hand, Cassar et al. [53], investigating mixed-parity sows assigned to be housed individually or in groups of 15 from the time of insemination for the 5 subsequent weeks, found no effect of grouping per se on farrowing rate or subsequent litter size.

An excellent study was presented in 2008 by Munsterhjelm et al. [54], investigating effects of housing on sow’s reproduction. Half of the dry sows were kept in stalls, and half were group-housed on 5 m^2^ deep litter straw bedding per sow at 28 days after mating. Stall housing was associated with signs of stress caused by lack of exercise and a rootable substrate. Behavioural indicators proposed a lower welfare level in stalled animals compared with group-housed ones. However, reproduction, in terms of weaning-to-oestrus interval and percentage of re-breedings, was negatively affected in the group-housed compared with the stalled sows.

#### ***Experimental “stress” studies***

To simulate stressful events after weaning and around pro-oestrus/oestrus, such as aggressive behaviour among sows after grouping/mixing, competitive situations at feeding and drinking, sows riding other sows, repeated injections of small doses of adrenocorticotropic hormone (ACTH) were given for approximately 48 hours to multiparous healthy sows around pro-oestrus and oestrus in a series of experiments. Some of the key findings in the ACTH-treated animals, besides several endocrine alterations, were that it was more common that the ovulation was disturbed [55]. When the sows were euthanized at 48 or 60 hours after ovulation, fewer oocytes/embryos were retrieved [56]. In an attempt to explain this finding, an *in vivo*–*in vitro* model was established where plasma from sows receiving ACTH or NaCl solution (controls) was used as a 10% supplement to media used for porcine *in vitro* embryo production. The conclusion from this *in vivo*–*in vitro* model was that the most stress-sensitive stage seems to be the short period precisely at ovulation, and that the male gamete seems to be most affected [57-59].

Another approach to simulate stressful events is food deprivation for 48 hours. In several studies, multiparous sows were food-deprived (FD), but had free access to drinking water, or were treated with ACTH for 48 hours after ovulation. There was a delayed embryonic cleavage rate and a decreased number of spermatozoa attached to zona pellucida (ZP) in FD sows, reflecting a change in the oviductal environment [60]. Postovulatory food deprivation has also been reported to delay the oviductal ova transport rate [61], which may be due to a prostaglandin-associated prolonged contraction of the isthmic muscle [62]. Adrenocorticotropic hormone had no effect on the oviductal transport rate of the embryos, but a negative effect was noted regarding the embryo development in terms of cleavage rate and a lower number of spermatozoa that had attached to the ZP compared with the controls [63,64].

When the treatments (FD or ACTH) were performed during days 13 and 14 of pregnancy, both FD and ACTH sows had increased levels of cortisol during the treatment period, but only FD sows had increased levels of progesterone and prostaglandin F 2 alpha (PGF_2α_) metabolite [65]. However, there were no effects on the total number of foetuses or foetal survival rate, observed on day 30 of pregnancy [66,67].

### Challenges and research needs

Our field and clinical observations suggest that by applying basic knowledge about the pig reproductive physiology related to mating, we can apply efficient batch-wise breeding in non-lactating, group-housed sows with competitive herd productivity. However, studies comparing different loose or group-housing systems show different outcomes for reproductive performance. This indicates that there is room for optimizing loose housing systems.

Experimental studies on reproduction related to grouping of sows have identified time of grouping, group size, the age/size of the sows grouped together, bedding, and feeding systems as important determinants. These experimental studies provide important data on how to design field studies to evaluate different loose and group-housing systems. Combination of experimental and field studies is a powerful approach in efforts to improve animal welfare, reproductive performance and economic productivity.

## Conclusion

It is possible to combine keeping sows in animal welfare friendly group housing according to EC Directive 2008/120/EC, with competitive reproductive performance and productivity. However, substantially increased research and development is needed to optimize these systems.

## Competing interests

The authors declare that they have no competing interests.

## Authors’ contributions

SE and UM planned the review. All authors contributed to the organization and preparation of the manuscript. SE, YS and UM finalized the manuscript. All authors approved the final version of the manuscript.
